# Prognostic value of stress echocardiography assessed by the ABCDE protocol

**DOI:** 10.1093/eurheartj/ehab493

**Published:** 2021-08-27

**Authors:** Quirino Ciampi, Angela Zagatina, Lauro Cortigiani, Karina Wierzbowska-Drabik, Jaroslaw D Kasprzak, Maciej Haberka, Ana Djordjevic-Dikic, Branko Beleslin, Alla Boshchenko, Tamara Ryabova, Nicola Gaibazzi, Fausto Rigo, Claudio Dodi, Iana Simova, Martina Samardjieva, Andrea Barbieri, Doralisa Morrone, Valentina Lorenzoni, Costantina Prota, Bruno Villari, Francesco Antonini-Canterin, Mauro Pepi, Clara Carpeggiani, Patricia A Pellikka, Eugenio Picano

**Affiliations:** Cardiology Division, Fatebenefratelli Hospital, Benevento, Italy; Cardiology Department, Saint Petersburg State University Hospital, Russian Federation; Cardiology Department, San Luca Hospital, Lucca, Italy; Chair of Cardiology, Bieganski Hospital, Medical University, Lodz, Poland; Chair of Cardiology, Bieganski Hospital, Medical University, Lodz, Poland; Department of Cardiology, SHS, Medical University of Silesia, Katowice, Poland; Cardiology Clinic, Clinical Center of Serbia, Medical School, University of Belgrade, Belgrade, Serbia; Cardiology Clinic, Clinical Center of Serbia, Medical School, University of Belgrade, Belgrade, Serbia; Cardiology Research Institute, Tomsk National Research Medical Centre of the Russian Academy of Sciences, Tomsk, Russian Federation; Cardiology Research Institute, Tomsk National Research Medical Centre of the Russian Academy of Sciences, Tomsk, Russian Federation; Cardiology Department, Parma University Hospital, Parma, Italy; Cardiology Department, Ospedale di Dolo-Venice, Venice, Italy; Cardiology Department, Ospedale di Cremona, Cremona, Italy; Cardiology Department, Heart and Brain Center of Excellence, University Hospital, Pleven, Sofia, Bulgaria; Cardiology Department, Heart and Brain Center of Excellence, University Hospital, Pleven, Sofia, Bulgaria; Noninvasive Cardiology, University Hospital, Parma, Italy; Cardiothoracic Department, University of Pisa, Italy; Institute of Management, Scuola Superiore Sant'Anna, Pisa, Italy; San Luca Hospital, Vallo della Lucania, Italy; Cardiology Division, Fatebenefratelli Hospital, Benevento, Italy; Highly Specialized Rehabilitation Hospital Motta di Livenza, Cardiac Prevention and Rehabilitation Unit, Treviso, Italy; Italian Society of Echocardiography and Cardiovascular Imaging, Milano, Italy; Italian Society of Echocardiography and Cardiovascular Imaging, Milano, Italy; Cardiology Division, Fondazione Cardiologica Monzino, Milano, Italy; Biomedicine Department, CNR, Institute of Clinical Physiology, Via Moruzzi 1, Building C- Room 130, 56124 Pisa, Italy; Department of Cardiovascular Medicine, Mayo Clinic, Rochester, MN, USA; Biomedicine Department, CNR, Institute of Clinical Physiology, Via Moruzzi 1, Building C- Room 130, 56124 Pisa, Italy

**Keywords:** Echocardiography, Outcome, Stress, Vulnerability

## Abstract

**Aim:**

The aim of this study was to assess the prognostic value of ABCDE-SE in a prospective, large scale, multicentre, international, effectiveness study. Stress echocardiography (SE) was recently upgraded to the ABCDE protocol: step A, regional wall motion abnormalities; step B, B lines; step C, left ventricular contractile reserve; step D, Doppler-based coronary flow velocity reserve in left anterior descending coronary artery; and step E, electrocardiogram-based heart rate reserve.

**Methods and results:**

From July 2016 to November 2020, we enrolled 3574 all-comers (age 65 ± 11 years, 2070 males, 58%; ejection fraction 60 ± 10%) with known or suspected chronic coronary syndromes referred from 13 certified laboratories. All patients underwent clinically indicated ABCDE-SE. The employed stress modality was exercise (*n* = 952, with semi-supine bike, *n* = 887, or treadmill, *n* = 65 with adenosine for step D) or pharmacological stress (*n* = 2622, with vasodilator, *n* = 2151; or dobutamine, *n* = 471). SE response ranged from score 0 (all steps normal) to score 5 (all steps abnormal). All-cause death was the only endpoint. Rate of abnormal results was 16% for A, 30% for B, 36% for C, 28% for D, and 37% for E steps. During a median follow-up of 21 months (interquartile range: 13–36), 73 deaths occurred. Global *X* ^2^ was 49.5 considering clinical variables, 50.7 after step A only (*P* = NS (not significant)) and 80.6 after B–E steps (*P* < 0.001 vs. step A). Annual mortality rate ranged from 0.4% person-year for score 0 up to 2.7% person-year for score 5.

**Conclusion:**

ABCDE-SE allows an effective prediction of survival in patients with chronic coronary syndromes.

## Graphical Abstract

**Figure:**
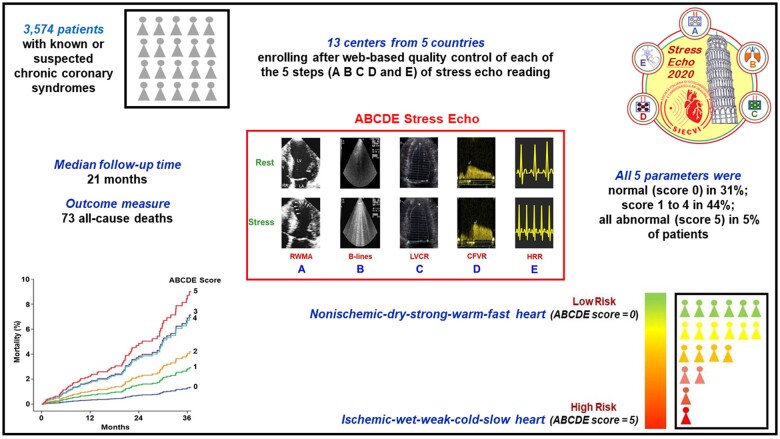
This study recruited 3,574 patients from 13 stress echo laboratories of 5 countries and shows the prognostic value of the new state-of-the art cardiac functional testing with stress echo ABCDE protocol: Step A for regional wall motion abnormalities; step B for B-lines obtained with 4-site simplified scan and lung ultrasound; step C for contractile reserve with volumetric echocardiography; step D for Doppler-based assessment of coronary flow reserve in mid-distal left anterior descending coronary artery; step E for EKG-based heart rate reserve.

**See page 3879 for the editorial comment on this article (doi:10.1093/eurheartj/ehab562)**

## Introduction

Stress echocardiography (SE) is an established diagnostic technique based on the detection of inducible myocardial ischaemia as regional wall motion abnormality (RWMA). This approach has a recognized clinical value but it seems unfit to describe the emerging complexity and heterogeneity of the individual patient vulnerability. Therefore, SE was recently upgraded to the ABCDE protocol:^1^ step A for RWMA, which is the only sign established in guidelines and recommendations;^2^ ^,^ ^3^ step B for B lines by lung ultrasound;^4^ ^,^ ^5^ step C for left ventricular contractile reserve (LVCR);^6^ ^,^ ^7^ step D for Doppler-based coronary flow velocity reserve (CFVR) in the left anterior descending coronary artery (LAD);^8^ and step E for imaging-independent heart rate reserve (HRR) based on the electrocardiogram (ECG).^9–11^ This approach applies to all types of physical or pharmacological stress modalities and allows a more integrated assessment of patient vulnerability above and beyond the detection of anatomic coronary artery disease (CAD). ABCDE-SE allows assessment of inducible myocardial ischaemia (step A), pulmonary congestion (step B), contractile reserve (step C), coronary microvascular dysfunction (step D), and cardiac sympathetic reserve (step E). The ABCDE-SE protocol is more likely to capture many potential sources of vulnerability of the patient with CAD^12^ ^,^ ^13^ than a simpler approach based only on RWMA.

The study hypothesis was that each pathophysiological variable may contribute independently and incrementally to the prognostic vulnerability in the individual patient and, therefore, the outcome would progressively worsen with the increasing number of abnormal steps during cardiac functional stress. To test this hypothesis, ABCDE-SE was performed in patients with chronic coronary syndromes, referred for clinically driven SE in accredited laboratories^14^ in the network of the international, multicentre, prospective SE 2020 study^15^ (*Graphical abstract*).

## Methods

### Study population

In this prospective study, we initially screened 4213 patients recruited from July 2016 to November 2020 by 13 certified laboratories of 5 countries (Bulgaria, Italy, Poland, Russian Federation, Serbia). Of these initial 4213, 639 did not complete the full protocol with missing information on step B (*n* = 281), step C (*n* = 18), or step D (*n* = 286). The main reasons were that the step was performed prior to specific training and certification for steps B and C, or the test was attempted but data were not obtained because of technical failure in step D. An additional 54 patients were lost to follow-up. The final study population included 3574 patients all studied with the full ABCDE protocol with outcome information.

The inclusion criteria were: (i) age >18 years; (ii) referral for known or suspected CAD; (iii) no severe primary valvular or congenital heart disease, or presence of prognosis-limiting comorbidities, such as advanced cancer, reducing life expectancy to <1 year; (iv) echocardiography of acceptable quality at rest and during stress; (v) willingness to give their written informed consent allowing scientific utilization of observational data, respectful of privacy rights; and (vi) recruitment in centres with systematic follow-up program.

All patients underwent SE testing as part of a clinically driven evaluation and according to the referring physician’s indications. Indications were in recommendation Class I or IIa according to the European Society of Cardiology^2^ and/or American Society of Echocardiography,^3^ falling into one of the three broad categories: initial test to diagnose obstructive CAD in patients with preserved ejection fraction, especially in patients with intermediate pre-test probability of disease (*n* = 1400, 39%); inconclusive exercise ECG or unable to exercise (*n* = 452, 13%); and prognosis assessment or re-assessment in stable CAD, with or without global left ventricular dysfunction (*n* = 1722, 48%).

Written informed consent was obtained from all patients before testing. The study protocol was reviewed and approved by the institutional ethics committees as a part of the SE 2020 study (148—Comitato Etico Lazio-1, 16 July 2016; ClinicalTrials.gov Identifier NCT03049995). The study was funded partly by the Italian National Research Council (Ageing project) and with travel grants of the Italian Society of Echocardiography and Cardiovascular Imaging with dedicated sessions during national meetings. No support from industry was received.

### Stress echocardiography

We used commercially available ultrasound machines. All patients underwent comprehensive transthoracic echocardiography at rest^16^ and underwent physical or pharmacological SE according to the protocol recommended by the European Association of Echocardiography^17^ and American Society of Echocardiography.^18^ The exercise protocol was semi-supine bike or post-treadmill. We used dipyridamole dose up to 0.86 mg/kg (over 4 or 6 min), dobutamine starting from 5 up to 40 μg/kg/min with atropine co-administration up to 1 mg, and adenosine up to 0.14 μg/kg/min over 6 min. In one centre (Belgrade), ABCE steps were acquired with treadmill exercise, and step D with adenosine (0.14 μg/kg/min in 2 min) in the same patients on the same day, 1 h after the treadmill test.

Criteria for terminating the test were severe chest pain, diagnostic ST-segment shift, excessive blood pressure increase (systolic blood pressure ≥240 mmHg, diastolic blood pressure ≥120 mmHg), limiting dyspnoea, maximal predicted heart rate, and significant arrhythmias. Echocardiographic imaging was performed from parasternal long- and short-axis views, and apical four- and two-chamber views, using conventional two-dimensional echocardiography. Contrast use was recommended in cases of suboptimal acoustic window, when ≥2 segments were not optimally visualized for 50% or more of endocardial length, and its use ranged from 0% to 40% in recruiting centres, depending on local lab policy and reimbursement issues. Anti-anginal drugs were usually not suspended before testing.

Step A included assessment of wall motion abnormalities and was performed in all patients. Wall motion score index (WMSI) was calculated in each patient at baseline and peak stress, in a 4-point score ranging from 1 (normal) to 4 (dyskinetic) in a 17-segment model of the left ventricle.

Step B of protocol included the assessment of B lines with lung ultrasound and the four-site simplified scan, from mid-axillary to mid-clavicular lines on the third intercostal space, each site scored from 0 (normal horizontal A lines) to 10 (white lung with coalescent B lines).^5^

Step C of protocol included the force-based assessment of LVCR as the stress/rest ratio of force, calculated as systolic blood pressure/end-systolic volume.^7^

Coronary flow velocity reserve (step D) was assessed during the standard SE examination using intermittent imaging of wall motion and LAD.^8^ Coronary flow in the mid-distal portion of the LAD was imaged from the low parasternal long-axis view and/or modified apical two-, three-, or four-chamber view under the guidance of colour Doppler flow mapping. All studies were digitally stored to simplify offline reviewing and measurements. At each time point, three optimal profiles of peak diastolic Doppler flow velocities were measured, and the results were averaged.

Heart rate reserve (step E) was calculated as the peak/rest heart rate from 12-lead ECG.^11^

**Figure 1:**
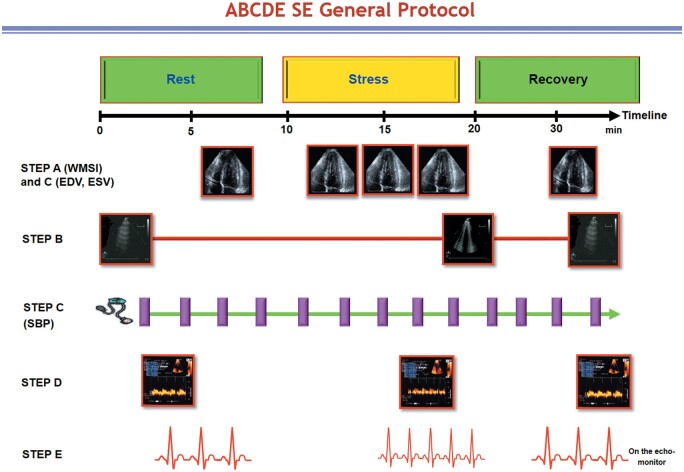
The ABCDE protocol. The completion of the test from preparation phase to written response takes ∼30 min. Recovery phase is optional, and advised in the presence of test positivity. Step A and step C require the very same images. Step E requires only one electrocardiogram lead. Step B can be completed in the early (<30 s) recovery phase. SBP, systolic blood pressure; SE, stress echocardiography.

All steps were performed by the same sonographer/cardiologist with the same transducer for cardiac, lung, and coronary scan—although occasionally a different high-frequency transducer was used for coronary flow. All steps were acquired at rest and peak stress. If needed, steps were repeated after 5 min in the recovery phase (*Figure 1*).

A detailed visual description of the scanning procedure is also available in a 9-min movie from the consortium (YouTube. ABCDE Stress Echo 2030: How I Make It. More easily done than said. Available at: https://www.youtube.com/watch?v=O4-5FjSF7ao, accessed 26 May 2021).

### Stress echocardiography positivity criteria

All positivity criteria were determined a priori.

The A criterion was considered positive in the presence of stress-induced RWMA (WMSI stress > rest), when at least two adjacent segments of the same vascular territory of the left ventricle showed an increment of at least one point of segmental score during SE.

The B criterion was considered positive in the presence of stress or rest B lines ≥2 units.^5^

The C criterion was considered positive in the presence of force-based LVCR ≤2.0 for exercise or dobutamine and ≤1.1 for vasodilators.^7^

The D criterion was considered positive in the presence of CFVR ≤2.0.^8^

The E criterion was considered positive in the presence of HRR <1.80 for exercise or dobutamine or <1.22 for dipyridamole or adenosine.^9–11^

As required by SE 2020 protocol, all readers had passed the quality control for each of the four imaging parameters upstream to starting patient recruitment.^15^

Stress echocardiography response was summarized with a score ranging from 0 to 5 as follows: score 0 (all ABCDE markers within normal limits) or scores 1–5, according to the number of abnormal steps (e.g. score 5 indicated that all 5 steps were abnormal).

### Data storage and analysis

The results for each test were entered in the data bank at the time of testing by each recruiting centre and sent monthly to the core lab with the electronic case report form with clinical data. After checking for internal consistency by trained technical staff, and double-checking with the centre for data verification on possibly inconsistent input, the data were added to the data bank and locked.

### Coronary angiography

Invasive coronary angiography (*n* = 1421) or non-invasive multidetector coronary angiography (*n* = 20) showing no CAD was available in 1441 patients. Coronary angiography was indicated by the referring physician based on symptoms, individual clinical characteristics, and non-invasive imaging results. Obstructive significant CAD was defined by a quantitatively assessed coronary diameter reduction ≥50% in the view showing the most severe stenosis. Images were read by experienced invasive cardiologists unaware of the SE results.

### Outcome data analysis

Deaths were identified from the national health service database. Non-deceased participants were contacted directly. Follow-up data were obtained from review of the patient’s hospital record, personal communication with the patient’s physician, review of the patient’s chart, a telephone interview with the patient or a patient’s close relative conducted by trained personnel, and a staff physician visiting the patients at regular intervals in the outpatient clinic. To avoid misclassification of the cause of death, overall death was considered. Assessors were blinded to clinical and SE results.

### Statistical analysis

Categorical data are expressed in terms of number of subjects and percentage while continuous data are expressed as mean ± standard deviation or median (minimum–maximum) depending on variable distribution. For continuous variables, differences among groups were tested with one-way analysis of variance followed by Bonferroni *post* *hoc* tests for between-group comparisons or Kruskal–Wallis followed by Mann–Whitney test using the Bonferroni correction, as appropriate. *χ* ^2^ test or Fisher’s exact test were used to compare the distribution of categorical variables among groups.

Survival was estimated using the Kaplan–Meier method and survival curves were compared by means of the log-rank test. Univariate Cox proportional hazards models were used to identify candidate predictors for selected endpoints. All variables with *P*-value <0.10 at univariate analysis were considered for inclusion in the multivariate Cox proportional hazards model. Collinearity was verified for all the models. We used the variance inflation factors to check the presence of collinearity and, for the multivariable model containing the positivity components, the overall mean variance inflation factor was 1.16. Moreover, values for all the variables were below 1.4, thus suggesting no evidence of collinearity in the model.

Non-proportionality of hazard was assessed using the Schoenfeld test. The incremental value of ABCDE-SE was evaluated comparing multivariable models with and without individual steps using global *X* ^2^ value to evaluate improvement of goodness-of-fit as well as continuous net reclassification index and integrated discrimination index to assess its incremental value. Statistical significance was set at *P*-value <0.05. All analyses were performed using STATA (STATACorp. Stata statistical software: Release 14. College Station, TX, USA).

## Results

The main clinical characteristics of the 3574 study patients are described in *Table 1*. The employed stress was exercise (*n* = 952, with semi-supine bicycle exercise: *n* = 887 or treadmill, *n* = 65), or pharmacological (*n* = 2622) with vasodilator (*n* = 2151; dipyridamole, *n* = 2112; adenosine, *n* = 39), or dobutamine (*n* = 471). Patients with more advanced ABCDE score (4 or 5), when compared to patients with lower scores, were more often hypertensives and studied under anti-ischaemic therapy with beta-blockers and nitrates (*Table 1*).

**Table 1:** Clinical characteristics of the patients

	All patients (*n* = 3574)	ABCDE score						*P*-value
		0 (*n* = 1114)	1 (*n* = 1064)	2 (*n* = 610)	3 (*n* = 349)	4 (*n* = 265)	5 (*n* = 172)	
Age (years)	65 ± 11	62 ± 11^a^ ^,^ ^b^ ^,^ ^c^ ^,^ ^d^	65 ± 11^c^	66 ± 11	68 ± 11^d^ ^,^ ^e^	66 ± 10	64 ± 9	<0.001
BSA (m^2^)	1.87 ± 0.22	1.86 ± 0.24^e^	1.87 ± 0.22	1.87 ± 0.24	1.85 ± 0.20	1.89 ± 0.19	1.92 ± 0.21	0.022
Sex (M/F)	2070 (58)	658 (59)	597 (56)	346 (57)	193 (55)	158 (60)	118 (69)	0.302
	1504 (42)	456 (41)	467 (44)	264 (43)	156 (45)	107 (40)	54 (31)	
Type of stress								<0.001
Exercise	952 (27)	113 (10)	241 (23)	171 (28)	101 (29)	178 (67)	148 (86)	
Vasodilator	2151 (60)	959 (86)	648 (61)	291 (48)	158 (45)	72 (27)	22 (13)	
Dobutamine	471 (13)	42 (4)	175 (16)	148 (24)	90 (26)	15 (6)	1 (1)	
Hypertension	2747 (77)	833 (75)	790 (74)	465 (75)	193 (76)	275 (79)	155 (90)	<0.001
Diabetes	846 (24)	212 (19)	244 (23)	167 (27)	111 (32)	67 (25)	45 (26)	<0.001
Previous PCI/CABG	1310 (40)	394 (37)	376 (43)	197 (43)	114 (46)	127 (52)	102 (62)	<0.001
Coronary angiography available	1441 (45)	432 (39)	411 (39)	287 (47)	161 (46)	104 (39)	46 (27)	<0.001
No CAD)	848 (59)	309 (71)	236 (64)	174 (61)	78 (48)	22 (21)	2 (4)	
1 vessel	373 (26)	108 (25)	112 (27)	66 (23)	43 (27)	33 (32)	11 (24)	
Multivessel	220 (15)	15 (4)	36 (9)	47 (16)	70 (25)	49 (47)	33 (72)	
History of myocardial infarction	928 (26)	259 (23)	275 (26)	150 (25)	96 (27)	77 (29)	71 (41)	<0.001
Beta-blockers	2179 (61)	529 (52)	635 (60)	393 (64)	245 (70)	193 (69)	134 (78)	<0.001
ACE-inhibitors/ARB	2343 (66)	651 (58)	678 (64)	415 (68)	256 (73)	198 (75)	145 (84)	<0.001
Nitrates	101 (3)	7 (1)	16 (2)	22 (4)	17 (5)	17 (6)	22 (13)	<0.001
Calcium channel blockers	633 (18)	146 (13)	169 (16)	114 (19)	89 (26)	71 (27)	44 (26)	<0.001
Diuretics	793 (22)	142 (11)	241 (23)	177 (29)	112 (32)	78 (29)	61 (35)	<0.001

Values are expressed as mean ± standard deviation or *n* (%).

ACE, angiotensin-converting enzyme; ARB, angiotensin receptor blocker; BSA, body surface area; CABG, coronary artery bypass graft; CAD, coronary artery disease; F, female; M, male; PCI, percutaneous coronary intervention.

a *P* < 0.05 vs. ABCDE score 1.

b *P* < 0.05 vs. ABCDE score 2.

c *P* < 0.05 vs. ABCDE score 3.

d *P* < 0.05 vs. ABCDE score 4.

e *P* < 0.05 vs. ABCDE score 5.

### Stress echocardiography findings

**Figure 2:**
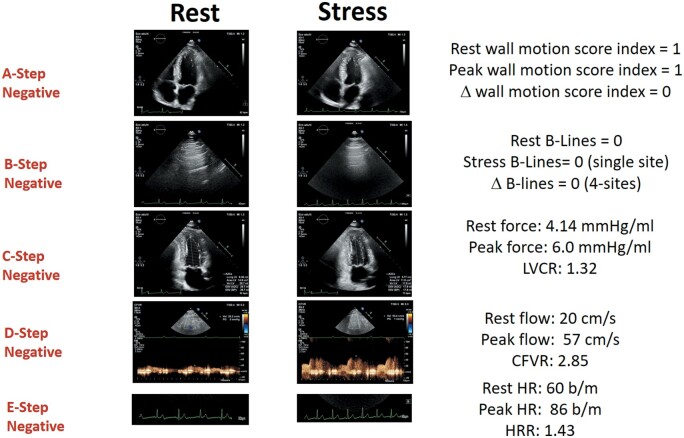
A normal ABCDE study. Left column: rest images. Right column, stress images. From top to bottom: A step: normal wall motion at rest and during stress; B step: normal A lines in lung ultrasound at rest and during stress; C step: reduced end-systolic volume and increased force during stress; D step: normal increase of pulsed-wave Doppler peak diastolic flow: 56/20 = 2.85; E step: normal heart rate increase on electrocardiogram (86/60 = 1.43). Stress modality: dipyridamole. CFVR, coronary flow velocity reserve; HR, hazard ratio; HRR, heart rate reserve; LVCR, left ventricular contractile reserve.

**Figure 3:**
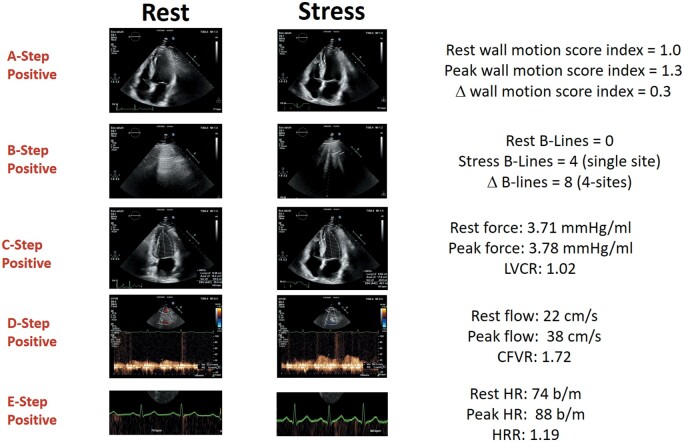
An abnormal ABCDE study. Left column: rest images. Right column, stress images. From top to bottom: A step: normal wall motion at rest and abnormal wall motion of apical region during stress; B step: no B lines at rest, four B lines in lung ultrasound during stress; C step: dilated end-systolic volume and blunted force reserve during stress; D step: reduced increase of pulsed-wave Doppler peak diastolic flow—38/22 = 1.72; E step: blunted heart rate on electrocardiogram (87/75 = 1.16). Stress modality: dipyridamole. CFVR, coronary flow velocity reserve; HR, hazard ratio; HRR, heart rate reserve; LVCR, left ventricular contractile reserve.

The main SE findings are described in *Table 2*. *Figures 2 and 3* show examples of ABCDE negativity and ABCDE positivity, respectively. Rate of abnormal results was 16% for A, 30% for B, 36% for C, 28% for D, and 37% for E steps.

**Table 2:** Haemodynamic, rest, and stress echocardiographic findings

	All patients (*n* = 3574)	ABCDE score						*P*-value
		0 (*n* = 1114)	1 (*n* = 1064)	2 (*n* = 610)	3 (*n* = 349)	4 (*n* = 265)	5 (*n* = 172)	
Rest WMSI	1.10 ± 0.23	1.05 ± 0.17^a^ ^,^ ^b^ ^,^ ^c^ ^,^ ^d^	1.07 ± 0.19^a^ ^,^ ^b^ ^,^ ^c^ ^,^ ^d^	1.11 ± 0.24^c^ ^,^ ^d^	1.15 ± 0.29^d^	1.18 ± 0.33	1.24 ± 0.36	<0.001
Stress WMSI	1.15 ± 0.31	1.04 ± 0.14^a^ ^,^ ^b^ ^,^ ^c^ ^,^ ^d^	1.06 ± 0.18^a^ ^,^ ^b^ ^,^ ^c^ ^,^ ^d^	1.12 ± 0.25^b^ ^,^ ^c^ ^,^ ^d^	1.22 ± 0.30^c^ ^,^ ^d^	1.53 ± 0.37^d^	1.82 ± 0.47	<0.001
ΔWMSI (stress–rest)	0.06 ± 0.21	−0.01 ± 0.07^a^ ^,^ ^b^ ^,^ ^c^ ^,^ ^d^	−0.01 ± 0.09^b^ ^,^ ^c^ ^,^ ^d^	0.01 ± 0.11^b^ ^,^ ^c^ ^,^ ^d^	0.06 ± 0.15^c^ ^,^ ^d^	0.35 ± 0.36^d^	0.58 ± 0.31	<0.001
A positivity	570 (16)	0	16 (2)	59 (10)	116 (33)	207 (78)	172 (100)	<0.001
B lines at rest	0.9 (0–35)	0.1 (0–1)	1.0 (0–32)	1.4 (0–30)	1.7 (0–34)	1.6 (0–20)	2.1 (0–35)	<0.001
B lines at peak	1.7 (0–40)	0.1 (0–1)^a^ ^,^ ^b^ ^,^ ^c^ ^,^ ^d^ ^,^ ^e^	1.4 (0–40)^a^ ^,^ ^b^ ^,^ ^c^ ^,^ ^d^	2.3 (0–24)^b^ ^,^ ^c^ ^,^ ^d^	3.1 (0–33)^c^ ^,^ ^d^	4.0 (0–40)^d^	6.3 (0–39)	<0.001
B positivity	1072 (30)	0	299 (28)	264 (43)	185 (53)	152 (57)	172 (100)	<0.001
DBP rest (mmHg)	78 ± 10	78 ± 10^d^	78 ± 10	79 ± 10	79 ± 9	79 ± 10	80 ± 11	0.011
DBP stress (mmHg)	78 ± 15	75 ± 14^a^ ^,^ ^b^ ^,^ ^c^ ^,^ ^d^ ^,^ ^e^	78 ± 15^c^ ^,^ ^d^	78 ± 15^c^ ^,^ ^d^	79 ± 15^d^	81 ± 15	83 ± 15	<0.001
SBP rest (mmHg)	132 ± 17	130 ± 15^a^ ^,^ ^b^ ^,^ ^c^ ^,^ ^d^ ^,^ ^e^	132 ± 17^a^ ^,^ ^b^	134 ± 18	136 ± 18	134 ± 17	135 ± 20	<0.001
SBP stress (mmHg)	142 ± 34	134 ± 30^a^ ^,^ ^b^ ^,^ ^c^ ^,^ ^d^ ^,^ ^e^	143 ± 35^c^ ^,^ ^d^	145 ± 35^c^ ^,^ ^d^	144 ± 34^c^ ^,^ ^d^	153 ± 32	156 ± 28	<0.001
EF rest (%)	60 ± 10	61 ± 6^a^ ^,^ ^b^	61 ± 8^b^	59 ± 16	58 ± 10	60 ± 11	61 ± 10	<0.001
EF stress (%)	69 ± 11	74 ± 8^a^ ^,^ ^b^ ^,^ ^c^ ^,^ ^d^ ^,^ ^e^	71 ± 9^a^ ^,^ ^b^ ^,^ ^c^ ^,^ ^d^	69 ± 11^b^ ^,^ ^c^ ^,^ ^d^	63 ± 12^c^ ^,^ ^d^	59 ± 12^d^	56 ± 11	<0.001
EDV rest (mL)	84 ± 29	77 ± 20^a^ ^,^ ^b^ ^,^ ^c^ ^,^ ^d^ ^,^ ^e^	83 ± 27^c^ ^,^ ^d^	87 ± 29^d^	89 ± 22^d^	98 ± 39	104 ± 37	<0.001
EDV stress (mL)	80 ± 29	72 ± 21^a^ ^,^ ^b^ ^,^ ^c^ ^,^ ^d^ ^,^ ^e^	78 ± 26^c^ ^,^ ^d^	83 ± 29^c^ ^,^ ^d^	84 ± 32^c^ ^,^ ^d^	96 ± 37	104 ± 37	<0.001
ESV rest (mL)	34 ± 19	30 ± 10^a^ ^,^ ^b^ ^,^ ^c^ ^,^ ^d^ ^,^ ^e^	35 ± 14^c^ ^,^ ^d^	36 ± 19^d^	38 ± 22	41 ± 29	43 ± 25	<0.001
ESV stress (mL)	26 ± 18	19 ± 8^a^ ^,^ ^b^ ^,^ ^c^ ^,^ ^d^ ^,^ ^e^	23 ± 13^a^ ^,^ ^b^ ^,^ ^c^ ^,^ ^d^	28 ± 17^b^ ^,^ ^c^ ^,^ ^d^	33 ± 21^c^ ^,^ ^d^	41 ± 28^d^	48 ± 27	<0.001
Force rest (mmHg/mL)	4.62 ± 1.96	4.86 ± 1.73^c^ ^,^ ^d^	4.63 ± 1.90^c^ ^,^ ^d^	4.60 ± 2.23^d^	4.51 ± 2.21	4.23 ± 1.87	4.04 ± 2.06	<0.001
Force peak (mmHg/mL)	7.39 ± 4.19	8.46 ± 3.93^a^ ^,^ ^b^ ^,^ ^c^ ^,^ ^d^	8.00 ± 4.24^a^ ^,^ ^b^ ^,^ ^c^ ^,^ ^d^	7.07 ± 4.56^b^ ^,^ ^c^ ^,^ ^d^	6.04 ± 3.86^c^ ^,^ ^d^	4.93 ± 2.68	4.27 ± 2.32	<0.001
LVCR	1.61 ± 0.72	1.77 ± 0.72^a^ ^,^ ^b^ ^,^ ^c^ ^,^ ^d^	1.76 ± 0.77^a^ ^,^ ^b^ ^,^ ^c^ ^,^ ^d^	1.56 ± 0.75^b^ ^,^ ^c^ ^,^ ^d^	1.35 ± 0.53^c^ ^,^ ^d^	1.17 ± 0.38	1.08 ± 0.29	<0.001
C positivity	1295 (26)	0	263 (25)	334 (55)	271 (78)	255 (96)	172 (100)	<0.001
LAD rest (cm/s)	26.8 ± 12.4	25.6 ± 7.4^c^ ^,^ ^d^	24.8 ± 11.0^b^ ^,^ ^c^ ^,^ ^d^	25.3 ± 13.3^c^ ^,^ ^d^	27.2 ± 12.4^c^ ^,^ ^d^	33.2 ± 17.6^d^	38.6 ± 21.2	<0.001
LAD stress (cm/s)	58.1 ± 24.0	65.5 ± 17.4^a^ ^,^ ^b^ ^,^ ^c^ ^,^ ^d^ ^,^ ^e^	59.0 ± 25.1^a^ ^,^ ^b^ ^,^ ^c^ ^,^ ^d^	53.8 ± 27.7	50.1 ± 23.3	49.7 ± 23.9	49.5 ± 25.5	<0.001
CFVR	2.25 ± 0.55	2.54 ± 0.36^a^ ^,^ ^b^ ^,^ ^c^ ^,^ ^d^ ^,^ ^e^	2.42 ± 0.44^a^ ^,^ ^b^ ^,^ ^c^ ^,^ ^d^	2.16 ± 0.45^b^ ^,^ ^c^ ^,^ ^d^	1.89 ± 0.45^c^ ^,^ ^d^	1.56 ± 0.42^d^	1.31 ± 0.28	<0.001
D positivity	994 (28)	0	111 (10)	221 (36)	245 (70)	245 (92)	172 (100)	<0.001
Heart rate rest (b.p.m.)	68 ± 11	65 ± 10^a^ ^,^ ^b^ ^,^ ^c^ ^,^ ^d^ ^,^ ^e^	68 ± 11^a^ ^,^ ^d^	70 ± 13^d^	69 ± 11^d^	70 ± 12^d^	73 ± 11	<0.001
Heart rate stress (b.p.m.)	103 ± 25	99 ± 21^a^ ^,^ ^e^	106 ± 28	108 ± 28^b^	102 ± 26	103 ± 20	102 ± 18	<0.001
HRR	1.53 ± 0.37	1.52 ± 0.31^d^ ^,^ ^e^	1.56 ± 0.43^b^ ^,^ ^c^ ^,^ ^d^	1.56 ± 0.42^b^ ^,^ ^c^	1.48 ± 0.34	1.48 ± 0.28	1.40 ± 0.19	<0.001
E positivity	1320 (37)	0	375 (35)	342 (56)	230 (66)	201 (76)	172 (100)	<0.001

Values are expressed as mean ± standard deviation, or *n* (%), except B-lines, expressed as median (range).

CFVR, coronary flow velocity reserve; DBP, diastolic blood pressure; EDV, end-diastolic volume; EF, ejection fraction; ESV, end-systolic volume; HRR, heart rate reserve; LAD, left anterior descending coronary artery; LVCR, left ventricular contractile reserve; SBP, systolic blood pressure; WMSI, wall motion score index.

a *P* < 0.05 vs. ABCDE score 2.

b *P* < 0.05 vs. ABCDE score 3.

c *P* < 0.05 vs. ABCDE score 4.

d *P* < 0.05 vs. ABCDE score 5.

e *P* < 0.05 vs. ABCDE score 1.

The positivity rate in various centres differed significantly, possibly due to different referral patterns and also because of the limited sample in some centres that determined a high degree of variability. In details, positivity rate ranged from 0% to 56% for step A, from 0% to 60% for step B, from 16% to 100% for step C, from 13% to 100% for step D, and from 8% to 86% for step E (Supplementary material online, *Table S1*). Each centre had its preferred stress modality. Stress modality by centre is shown in Supplementary material online, *Table S2*.

All five parameters were normal (score 0) in 1114 patients (31%), all five were abnormal (score 5) in 172 patients (5%). The abnormal score was 1 in 1064 (30%), 2 in 610 (17%), 3 in 349 (10%), and 4 in 265 (7%) patients.

**Figure 4:**
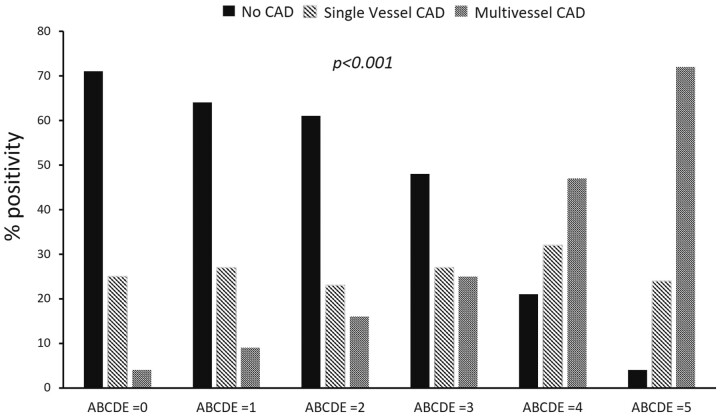
ABCDE-stress echocardiography results and coronary angiography data. The percentage of patients with normal coronary arteries (no coronary artery disease), one-vessel coronary artery disease, and multivessel or left main coronary artery disease in the six subsets identified with ABCDE score from 0 to 5. *Inter-group significance. CAD, coronary artery disease.

In the 1441 patients with coronary angiography available, 848 patients (59%) showed no CAD, 373 patients (26%) showed one-vessel CAD, and 220 patients (15%) showed multivessel CAD. ABCDE score increased progressively with increasing extent of CAD (*Figure 4*).

### Outcome data results

**Figure 5:**
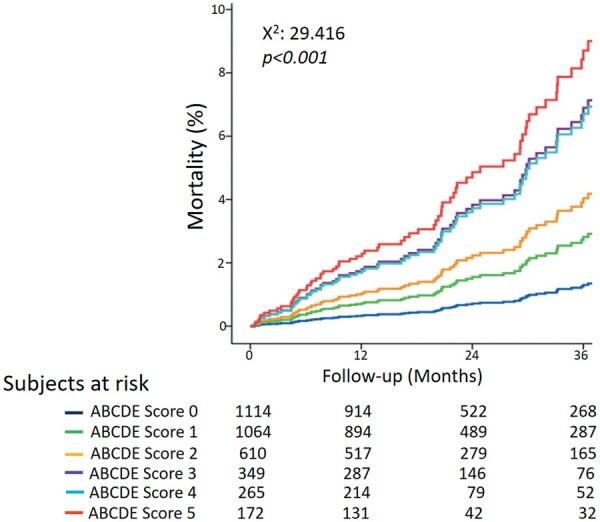
Survival curves based on ABCDE score. Survival is worse in patients with ABCDE score 5 and best in patients with ABCDE score 0.

During a median follow-up of 21 months (interquartile range: 13–36), 73 deaths occurred. In the univariable analysis, predictors of all-cause mortality were step B [hazard ratio (HR) 2.621, 95% confidence interval (CI) 1.654–4.152, *P* < 0.001], step D (HR 2.578, 95% CI 1.624–4.093, *P* < 0.001), and step E (HR 2.955, 95% CI 1.848–4.725; *P* < 0.001), but not step A (HR 1.333, 95% CI 0.731–2.430; *P* = 0.349) and step C (HR 1.581, 95% CI 0.997–2.506; *P* = 0.051) (*Table 3*). In the multivariable analysis, ABCDE-SE was an independent predictor of mortality with scores 3 (HR 3.472, 95% CI 1.483–8.135; *P* = 0.004), 4 (HR 4.045, 95% CI 1.595–10.259; *P* = 0.004), and 5 (HR 5.678, 95% CI 2.106–15.313; *P* < 0.001). Annual mortality rate ranged from 0.4% person-year for score 0 up to 2.7% person-year for score 5. Survival was worse in patients with ABCDE score 5 and best in patients with ABCDE score 0 (*Figure 5*).

**Table 3:** Predictors of all-cause mortality

Variables	Univariable Cox regression analysis		Multivariable Cox regression analysis	
	HR (95% CI)	*P*-value	HR (95% CI)	*P*-value
Age (years)	1.074 (1.047–1.101)	<0.001	1.065 (1.038–1.092)	<0.001
Male sex	1.968 (1.167–3.321)	0.011	1.964 (1.162–3.320)	0.012
Type of stress	0.759 (0.101–5.703)	0.789		
Exercise (reference)	1			
Dipyridamole	0.859 (0.495–1.491)	0.588		
Dobutamine	0.741 (0.332–1.654)	0.464		
Adenosine	1.317 (0.175–9.890)	0.789		
Hypertension (%)	1.547 (0.849–2.820)	0.154		
Beta-blockers therapy (%)	1.016 (0.631–1.637)	0.948		
Diabetes (%)	2.562 (1.613–4.070)	<0.001	1.946 (1.221–3.103)	0.005
Prior MI	1.280 (0.785–2.087)	0.322		
Reduced EF (<50%)	1.866 (1.041–3.343)	0.03		
A positivity	1.333 (0.731–2.430)	0.349		
B positivity	2.621 (1.654–4.152)	<0.001		
C positivity	1.581 (0.997–2.506)	0.051		
D positivity	2.578 (1.624–4.093)	<0.001		
E positivity	2.955 (1.848–4.725)	<0.001		
ABCDE score 0	1		1	
ABCDE score 1	2.182 (0.987–4.824)	0.054	1. 798 (0.811–3.988)	0.149
ABCDE score 2	2.974 (1.301–6.797)	0.010	2.187 (0.934–5.043)	0.066
ABCDE score 3	5.438 (2.352–12.573)	<0.001	3.472 (1.483–8.135)	0.004
ABCDE score 4	5.289 (2.095–13.354)	<0.001	4.045 (1.595–10.259)	0.004
ABCDE score 5	6.923 (2.573–18.625)	<0.001	5.678 (2.106–15.313)	<0.001

CI, confidence interval; EF, ejection fraction; HR, hazard ratio; MI, myocardial infarction.

**Figure 6:**
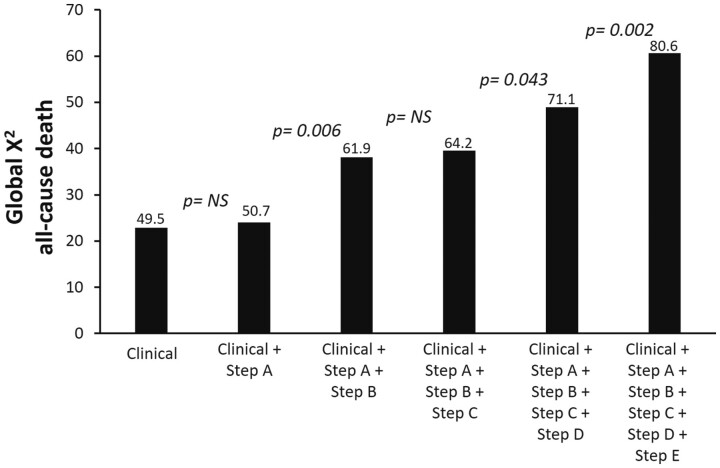
Incremental prognostic value of ABCDE positivity. The incremental value of ABCDE on global *X* ^2^ for predicting all-cause death was non-significant for step A (after considering clinical variables) and step C (after considering step B) and significant for steps B, D, and E. LVEF, left ventricular ejection fraction. NS, •••.

In the incremental analysis, global *X* ^2^ of clinical model for the prediction of death increased from 49.5 considering clinical variables positive at univariate analysis to 50.7 after step A only (*P* = NS), and 80.6 after BCDE steps (*P* < 0.001 vs. step A). The increase over clinical evaluation was not significant after step A (after considering clinical variables) and step C (after considering step B) and significant for steps B, D, and E (*Figure 6*).

Adding ABCDE-SE, risk reclassification was significantly improved with net reclassification index: 23.4% (95% CI 0.26–46.5%; *P* = 0.048). Integrated discrimination index was 0.51% (95% CI 0.01–0.94%; *P* = 0.020).

## Discussion

A higher ABCDE-SE score indicates a less benign outcome. The risk is extremely low when all biomarkers are normal. In the individual biomarker analysis, the more established step A is also the less useful, outperformed by steps B, C, D and E for predicting all-cause death. Prognostically meaningful indices such as HRR and B lines are also very simple to obtain and analyse. Step B requires basic 2D technology and a minimal training, acquisition, and analysis time. Step E is imaging independent and automatically read in real time on the monitor of the echocardiographic machine. The value of ABCDE approach is coherent with the current understanding of the pathophysiological complexity of CAD. The individual patient with CAD may have multiple vulnerabilities, which are ignored, and missed, by the conventional step A approach. With ABCDE, the focus is shifted from coronary stenoses to the patient as a whole, with a simultaneous assessment of multiple vulnerabilities, from coronary microvascular dysfunction^19^ to cardiac autonomic unbalance.^20^ It is conceivable that an SE-driven tailored therapy might be effective in improving prognosis through targeted efforts against the abnormal steps, which may trigger specific countermeasures. ABCDE-SE changes the binary step A response in a refined stratification of the overall level of risk, with a better identification of the prevailing phenotype coming into play in a specific patient.

### Comparison with previous studies

The findings of the present study are consistent with previous reports showing the diagnostic and prognostic values of step B,^5^ ^,^ ^21^ step C,^6^ step D,^8^ ^,^ ^22^ and step E^9–11^ separately performed. The low positivity rate with ischaemia by SE or myocardial perfusion imaging has been observed in recent years, possibly due to a change in population characteristics.^23^ The prognostic value of various steps has also been extensively shown to be additive to stress-induced RWMA for step B,^5^ step C,^6^ step D,^8^ and step E.^24^ Step D offers independent information beyond steps A, C, and E.^8^ ^,^ ^24^ The results of the present study confirm and extend previous evidence since all steps were simultaneously assessed in the same population allowing a separate analysis for all-cause death.

The test positivity based on RWMA did not predict mortality.^6^ Most patients are now studied under therapy that masks inducible ischaemia. In addition, step A is the only parameter embedded in general cardiology guidelines^2^ and is utilized by referring cardiologists to decide ischaemia-driven revascularization to improve symptoms and possibly, in some subsets, survival.

### Clinical implications

Cardiac functional testing with ABCDE-SE allows gaining of a comprehensive insight on patient vulnerability still with an extraordinarily simple and feasible test with low cost, minimal risk, zero radiation, and near-zero environmental impact. Therefore, it has the ideal features of economic, radiological, and environmental sustainabilities that are especially important for health care in the COVID-19 era. The protocol can be applied with all stresses, and this is especially important since exercise can be easily replaced with pharmacological test in periods of intense viral epidemiological pressure when pharmacological stress is preferred over exercise because of added safety concerns and needed personal protective equipment during exercise.^25^

The use of the ABCDE protocol is not mainly intended for the primary detection of CAD, since epicardial artery stenosis is the target of step A. However, the presence of advanced ABCDE score, even with normal step A, points to more extensive anatomic CAD in patients with chronic coronary syndromes. Patients with chest pain and CAD may show pulmonary congestion, reduced LVCR, a reduced CFVR, and blunted HRR in the absence of RWMA. A similar pattern can be found, even more frequently, in patients with chest pain with angiographically normal coronary arteries. The reassuring view of the classical ischaemic cascade with RWMA as the early and often only marker of inducible ischaemia is clearly challenged by these findings.

The main applications of ABCDE-SE are the identification of functional mechanisms of disease and symptoms, risk stratification, guide to therapy, or objective assessment of therapy efficacy. Each step identifies a specific phenotype, a biomarker of risk, and a potential selective target of tailored therapy.

An abnormal step A indicates anti-ischaemic therapy with beta-blockers, calcium channel blockers or nitrates, and possibly revascularization.^2^ Diuretic therapy is recommended in patients with pulmonary congestion,^2^ which is optimally identified and quantified with an abnormal step B after stress. Angiotensin-converting enzyme inhibitors are recommended in patients with asymptomatic left ventricular dysfunction after myocardial infarction,^2^ but dysfunction is better identified with contractile reserve in step C. A selectively abnormal step D suggests the presence of coronary microvascular disease, and statins are recommended.^2^ Abnormal step E implies a reduced cardiac sympathetic reserve, potentially targetable with a variety of possible therapies reducing the overactive sympathetic nervous system through the blockade of the beta-adrenergic or renin-angiotensin-aldosterone systems, or neuromodulation therapy aimed to restore autonomic balance through a sympatho-modulatory intervention, such as for instance renal denervation.^26^ The overall global ABCDE risk score may be important to titrate therapy for event prevention.

### Study limitations

Results were accessible to the referring physician, but the information beyond step A is unlikely to have affected subsequent decision-making since no evidence was available to support a change in management based on steps B–E.

Stress echocardiography does not tolerate improvisation, and a dedicated training is required to start ABCDE activity. However, the additional training and technology requirements are minimal compared to standard conventional SE, and analysis of wall motion and volumes is rapidly becoming operator independent and objective in the era of artificial intelligence.^27^ The most difficult technical hurdle is the acquisition of step D for LAD flow. This parameter was recommended with vasodilator stress echo since 2009^17^ and is already present in the 2019 European Society of Cardiology guidelines on chronic coronary syndromes for the assessment of coronary microvascular dysfunction in patients with ischaemia and normal coronary arteries.^2^ The next step for those willing to try and start is to put together this information in the clinical arena and to build their own experience in their own patients.

The original statistical plan was to include 5000 patients with 3-year follow-up for mortality analysis, but as already described for many studies, COVID-19 pandemic abruptly slowed the recruitment everywhere at the beginning of 2020,^28^ which was the planned last year of the SE 2020 study.

The outcome endpoint was all-cause death, which is a methodologically sound and clinically meaningful indicator but does not allow a separate analysis for different causes of death. In practice, the endpoint death is extremely robust, while the adjudication of cardiac or non-cardiac death, and even more sudden or non-sudden cardiac death, can be more problematic.^29^ More granular endpoints may be preferentially predicted by individual biomarkers: step A predicts unstable angina;^3^ ^,^ ^17^ step B predicts acute decompensated heart failure;^5^ step C predicts progression of dyspnoea class and left ventricular dilatation;^6^ step D predicts myocardial infarction;^8^ and step E predicts electrical instability and arrhythmias.^30^

The echocardiographic images were not adjudicated by a core lab but at each recruiting centre. This allowed a substantial sparing of resources and was also a methodological prerequisite for an effectiveness study, mirroring real-life conditions with unselected patients. A mandatory web-based quality control was implemented for conventional and innovative parameters upstream to patient recruitment since the volume of activity is necessary but not sufficient to ensure the quality of reading.^14^

The percentage of exercise, vasodilator, and dobutamine is different in the different subgroups, and this may influence at least the extent of positivity, although we found that the rate of positivity was similar with different stresses when stress-specific cut-off values are used.^5^ The score 0–5 underuses the potential of stratification since every single letter used to build the score, from A to E, is analysed as a binary (positive vs. negative) response but is amenable to further titration of severity, from mild to moderate to severe impairment. These aspects are certainly important and will likely be addressed in the future with larger sample sizes, longer follow-up, and artificial intelligence-based data analysis as planned in the SE 2030 study.

The overall score generated by the ABCDE protocol is useful to identify the level of global risk, with higher scores requiring more aggressive treatment and closer surveillance. On top of this methodological and conceptual framework, further steps can be added in specific patients, from mitral regurgitation (step F) for valvular or ischaemic heart disease to step G (for gradients) in hypertrophic cardiomyopathy up to step R (for right ventricular function) in valvular and some forms of congenital heart disease.

## Conclusions

ABCDE-SE allows assessment of multiple different vulnerabilities of the patient above and beyond epicardial artery stenosis. Vulnerabilities include pulmonary congestion, reduced contractile reserve, coronary microvascular dysfunction, and cardiac autonomic unbalance. This spectrum of phenotypes identifies the individual risk more accurately than inducible ischaemia and RWMA. Each step is important in modulating outcome and is a potential selective therapeutic target, paving the way to personalized assessment of risk and tailored therapy approaches.

## Supplementary material

Supplementary material is available at *European Heart Journal* online.

## Funding

The study was partially funded by CNR-MIUR (National Research Council, Italian Ministry of University and Research) Ageing subproject (Progetto P001328, Progetto di Interesse-Invecchiamento).

**Conflict of interest:** none declared.

## Data availability

The data presented in this study are available on request from the principal investigator (QC). The data are not publicly available due to privacy.

## Supplementary Material

ehab493_Supplementary_Data

## References

[1] Picano E, Zagatina A, Wierzbowska-Drabik K, Borguezan Daros C, D’Andrea A, Ciampi Q. Sustainability and versatility of the ABCDE protocol for stress echocardiography. J Clin Med 2020;9:3184. 10.3390/jcm9103184 PMC7601661 33008112

[2] Knuuti J, Wijns W, Saraste A, Capodanno D, Barbato E, Funck-Brentano C, Prescott E, Storey RF, Deaton C, Cuisset T, Agewall S, Dickstein K, Edvardsen T, Escaned J, Gersh BJ, Svitil P, Gilard M, Hasdai D, Hatala R, Mahfoud F, Masip J, Muneretto C, Valgimigli M, Achenbach S, Bax JJ; ESC Scientific Document Group. 2019 ESC Guidelines for the diagnosis and management of chronic coronary syndromes. Eur Heart J 2020;41:407–477. 31504439 10.1093/eurheartj/ehz425

[3] Pellikka PA, Arruda-Olson A, Chaudhry FA, Chen MH, Marshall JE, Porter TR, Sawada S. Guidelines for performance, interpretation, and application of stress echocardiography in ischemic heart disease: from the American Society of Echocardiography. J Am Soc Echocardiogr 2020;33:1–41.e8. 31740370 10.1016/j.echo.2019.07.001

[4] Scali MC, Cortigiani L, Simionuc A, Gregori D, Marzilli M, Picano E. Exercise-induced B-lines identify worse functional and prognostic stage in heart failure patients with depressed left ventricular function. Eur J Heart Fail 2017;19:1468–1478. 28198075 10.1002/ejhf.776

[5] Scali MC, Zagatina A, Ciampi Q, Cortigiani L, D’Andrea A, Daros CB, Zhuravskaya N, Kasprzak JD, Wierzbowska-Drabik K, Luis de Castro E Silva Pretto J, Djordjevic-Dikic A, Beleslin B, Petrovic M, Boskovic N, Tesic M, Monte I, Simova I, Vladova M, Boshchenko A, Vrublevsky A, Citro R, Amor M, Vargas Mieles PE, Arbucci R, Merlo PM, Lowenstein Haber DM, Dodi C, Rigo F, Gligorova S, Dekleva M, Severino S, Lattanzi F, Morrone D, Galderisi M, Torres MAR, Salustri A, Rodrìguez-Zanella H, Costantino FM, Varga A, Agoston G, Bossone E, Ferrara F, Gaibazzi N, Celutkiene J, Haberka M, Mori F, D'Alfonso MG, Reisenhofer B, Camarozano AC, Miglioranza MH, Szymczyk E, Wejner-Mik P, Wdowiak-Okrojek K, Preradovic-Kovacevic T, Bombardini T, Ostojic M, Nikolic A, Re F, Barbieri A, Di Salvo G, Merli E, Colonna P, Lorenzoni V, De Nes M, Paterni M, Carpeggiani C, Lowenstein J, Picano E; Stress Echo 2020 Study Group of the Italian Society of Echocardiography and Cardiovascular Imaging. Lung ultrasound and pulmonary congestion during stress echocardiography. JACC Cardiovasc Imaging 2020;13:2085–2095. 32682714 10.1016/j.jcmg.2020.04.020

[6] Cortigiani L, Huqi A, Ciampi Q, Bombardini T, Bovenzi F, Picano E. Integration of wall motion, coronary flow velocity, and left ventricular contractile reserve in a single test: prognostic value of vasodilator stress echocardiography in patients with diabetes. J Am Soc Echocardiogr 2018;31:692–701. 29625884 10.1016/j.echo.2017.11.019

[7] Bombardini T, Zagatina A, Ciampi Q, Cortigiani L, D'andrea A, Borguezan Daros C, Zhuravskaya N, Kasprzak JD, Wierzbowska-Drabik K, De Castro E, Silva Pretto JL, Djordjevic-Dikic A, Beleslin B, Petrovic M, Boskovic N, Tesic M, Ip M, Simova I, Vladova M, Boshchenko A, Ryabova T, Citro R, Amor M, Vargas Mieles PE, Arbucci R, Dodi C, Rigo F, Gligorova S, Dekleva M, Severino S, Torres MA, Salustri A, Rodrìguez-Zanella H, Costantino FM, Varga A, Agoston G, Bossone E, Ferrara F, Gaibazzi N, Rabia G, Celutkiene J, Haberka M, Mori F, D'alfonso Mg Reisenhofer B, Camarozano AC, Salamé M, Szymczyk E, Wejner-Mik P, Wdowiak-Okrojek K, Preradovic TK, Lattanzi F, Morrone D, Scali MC, Ostojic M, Nikolic A, Re F, Barbieri A, Di Salvo G, Colonna P, De Nes M, Paterni M, Merlo PM, Lowenstein J, Carpeggiani C, Gregori D, Picano E; Stress Echo 2020 Study Group of the Italian Society of Echocardiography and Cardiovascular Imaging. Feasibility and value of two-dimensional volumetric stress echocardiography. Minerva Cardioangiol 2020; doi:10.23736/S0026-4725.20.05304-9.

[8] Ciampi Q, Zagatina A, Cortigiani L, Gaibazzi N, Borguezan Daros C, Zhuravskaya N, Wierzbowska-Drabik K, Kasprzak JD, de Castro E Silva Pretto JL, D'Andrea A, Djordjevic-Dikic A, Monte I, Simova I, Boshchenko A, Citro R, Amor M, Merlo PM, Dodi C, Rigo F, Gligorova S, Dekleva M, Severino S, Lattanzi F, Scali MC, Vrublevsky A, Torres MAR, Salustri A, Rodrìguez-Zanella H, Costantino FM, Varga A, Bossone E, Colonna P, De Nes M, Paterni M, Carpeggiani C, Lowenstein J, Gregori D, Picano E; Stress Echo 2020 Study Group of the Italian Society of Echocardiography and Cardiovascular Imaging. Functional, anatomical, and prognostic correlates of coronary flow velocity reserve during stress echocardiography. J Am Coll Cardiol 2019;74:2278–2291. 31672185 10.1016/j.jacc.2019.08.1046

[9] Elhendy A, Mahoney DW, Khandheria BK, Burger K, Pellikka PA. Prognostic significance of impairment of heart rate response to exercise. Impact of left ventricular function and myocardial ischemia. J Am Coll Cardiol 2003;42:823–830. 12957427 10.1016/s0735-1097(03)00832-5

[10] Chaowalit N, Mc Cully RB, Callahan MJ, Mookadam F, Bailey KM, Pellikka PA. Outcomes after normal dobutamine stress echocardiography and predictors of adverse events: long-term follow-up of 3014 patients. Eur Heart J 2006;27:3039–3044. 17132654 10.1093/eurheartj/ehl393

[11] Cortigiani L, Carpeggiani C, Landi P, Raciti M, Bovenzi F, Picano E. Usefulness of blunted heart rate reserve as an imaging-independent prognostic predictor during dipyridamole-echocardiography test. Am J Cardiol 2019;124:972–977. 31324358 10.1016/j.amjcard.2019.06.017

[12] Marzilli M, Merz CN, Boden WE, Bonow RO, Capozza PG, Chilian WM, DeMaria AN, Guarini G, Huqi A, Morrone D, Patel MR, Weintraub WS. Obstructive coronary atherosclerosis and ischemic heart disease: an elusive link! J Am Coll Cardiol 2012;60:951–956. 22954239 10.1016/j.jacc.2012.02.082

[13] Arbab-Zadeh A, Fuster V. From detecting the vulnerable plaque to managing the vulnerable patient: JACC state-of-the-art review. J Am Coll Cardiol 2019;74:1582–1593. 31537269 10.1016/j.jacc.2019.07.062

[14] Ciampi Q, Picano E, Paterni M, Daros CB, Simova I, de Castro E Silva Pretto JL, Scali MC, Gaibazzi N, Severino S, Djordjevic-Dikic A, Kasprzak JD, Zagatina A, Varga A, Lowenstein J, Merlo PM, Amor M, Celutkiene J, Perez JE, Di Salvo G, Galderisi M, Mori F, Costantino MF, Massa L, Dekleva M, Chaves DQ, Trambaiolo P, Citro R, Colonna P, Rigo F, Torres MAR, Monte I, Stankovic I, Neskovic A, Cortigiani L, Re F, Dodi C, D'Andrea A, Villari B, Arystan A, De Nes M, Carpeggiani C; Stress Echo 2020 Study Group of the Italian Society of Cardiovascular Echography. Quality control of regional wall motion analysis in Stress Echo 2020. Int J Cardiol 2017;249:479–485. 28986062 10.1016/j.ijcard.2017.09.172

[15] Picano E, Ciampi Q, Citro R, D'Andrea A, Scali MC, Cortigiani L, Olivotto I, Mori F, Galderisi M, Costantino MF, Pratali L, Di Salvo G, Bossone E, Ferrara F, Gargani L, Rigo F, Gaibazzi N, Limongelli G, Pacileo G, Andreassi MG, Pinamonti B, Massa L, Torres MA, Miglioranza MH, Daros CB, de Castro E Silva Pretto JL, Beleslin B, Djordjevic-Dikic A, Varga A, Palinkas A, Agoston G, Gregori D, Trambaiolo P, Severino S, Arystan A, Paterni M, Carpeggiani C, Colonna P. Stress Echo 2020: the international stress echo study in ischemic and non-ischemic heart disease. Cardiovasc Ultrasound 2017;15:3. 28100277 10.1186/s12947-016-0092-1 PMC5242057

[16] Lang RM, Badano LP, Mor-Avi V, Afilalo J, Armstrong A, Ernande L, Flachskampf FA, Foster E, Goldstein SA, Kuznetsova T, Lancellotti P, Muraru D, Picard MH, Rietzschel ER, Rudski L, Spencer KT, Tsang W, Voigt J-U. Recommendations for cardiac chamber quantitation by echocardiography: an update from the American Society of Echocardiography and the European Association of Cardiovascular Imaging. J Am Soc Echocardiogr 2015;28:1–39.e14. 25559473 10.1016/j.echo.2014.10.003

[17] Sicari R, Nihoyannopoulos P, Evangelista A, Kasprzak J, Lancellotti P, Poldermans D, Voigt JU, Zamorano JL; European Association of Echocardiography. Stress echocardiography expert consensus statement. European Association of Echocardiography (EAE) (a registered branch of the ESC). Eur Heart J 2009;30:278–289. 19001473 10.1093/eurheartj/ehn492

[18] Lancellotti P, Pellikka PA, Budts W, Chaudhry FA, Donal E, Dulgheru R, Edvardsen T, Garbi M, Ha JW, Kane GC, Kreeger J, Mertens L, Pibarot P, Picano E, Ryan T, Tsutsui JM, Varga A. The clinical use of stress echocardiography in non-ischaemic heart disease: recommendations from the European Association of Cardiovascular Imaging and the American Society of Echocardiography. J Am Soc Echocardiogr 2017;30:101–138. 28164802 10.1016/j.echo.2016.10.016

[19] Crea F, Camici PG, Bairey Merz CN. Coronary microvascular dysfunction: an update. Eur Heart J 2014;35:1101–1111. 24366916 10.1093/eurheartj/eht513 PMC4006091

[20] Brubaker PH, Kitzman DW. Chronotropic incompetence. Causes, consequences and management. Circulation 2011;123:1010–1020. 21382903 10.1161/CIRCULATIONAHA.110.940577 PMC3065291

[21] Wiley BM, Luoma CE, Olgun Kucuk H, Padang R, Kane GC, Pellikka PA. Lung ultrasound during stress echocardiography aids the evaluation of valvular heart disease severity. JACC Cardiovasc Imaging 2020;13:866–872. 31422148 10.1016/j.jcmg.2019.06.026

[22] Takeuchi M, Lodato JA, Furlong KT, Lang RM, Yoshikawa J. Feasibility of measuring coronary flow velocity and reserve in the left anterior descending coronary artery by transthoracic Doppler echocardiography in a relatively obese American population. Echocardiography 2005;22:225–232. 15725157 10.1111/j.0742-2822.2005.04004.x

[23] Peteiro J, Bouzas-Mosquera A, Broullon J, Sanchez-Fernandez G, Perez-Cebey L, Yañez J, Martinez D, Vazquez-Rodriguez JM. Outcome by exercise echocardiography in patients with low pretest probability of coronary artery disease. J Am Soc Echocardiogr 2016;29:736–744. 27112362 10.1016/j.echo.2016.03.001

[24] Cortigiani L, Ciampi Q, Carpeggiani C, Bovenzi F, Picano E. Prognostic value of heart rate reserve is additive to coronary flow velocity reserve during dipyridamole stress echocardiography. Arch Cardiovasc Dis 2020;113:244–‐251. 32241716 10.1016/j.acvd.2020.01.005

[25] Zoghbi WA, DiCarli MF, Blankstein R, Choi AD, Dilsizian V, Flachskampf FA, Geske JB, Grayburn PA, Jaffer FA, Kwong RY, Leipsic JA, Marwick TH, Nagel E, Nieman K, Raman SV, Salerno M, Sengupta PP, Shaw LJ, Chandrashekhar YS; ACC Imaging Council. Multimodality cardiovascular imaging in the midst of the COVID-19 pandemic: ramping up safely to a new normal. JACC Cardiovasc Imaging 2020;13:1615–1626. 32646721 10.1016/j.jcmg.2020.06.001 PMC7290215

[26] Hanna P, Shivkumar K, Ardell JL. Calming the nervous heart: autonomic therapies in heart failure. Card Fail Rev 2018;4:92–98. 30206483 10.15420/cfr.2018.20.2 PMC6125704

[27] Dey D, Slomka PJ, Leeson P, Comaniciu D, Shrestha S, Sengupta PP, Marwick TH. Artificial intelligence in cardiovascular imaging: JACC state-of-the-art review. J Am Coll Cardiol 2019;73:1317–1335. 30898208 10.1016/j.jacc.2018.12.054 PMC6474254

[28] Gaudino M, Arvind V, Hameed I, Di Franco A, Spadaccio C, Bhatt DL, Bagiella E. Effects of the COVID pandemic on active non-COVID clinical trials. J Am Coll Cardiol 2020;76:1605–1606. 32745501 10.1016/j.jacc.2020.07.051 PMC7834205

[29] Lauer M, Blackstone E, Young J, Topol E. Cause of death in clinical research: time for reassessment? J Am Coll Cardiol 1999;34:618–620. 10483939 10.1016/s0735-1097(99)00250-8

[30] Freeman JV, Dewey FE, Hadley DM, Myers J, Froelicher VF. Autonomic nervous system interaction with the cardiovascular system during exercise. Prog Cardiovasc Dis 2006;48:342–362. 16627049 10.1016/j.pcad.2005.11.003

