# Supplementary material for: Prognostic value of stress echocardiography assessed by the ABCDE protocol
Source: Eur Heart J. 2021 Aug 27;42(37):3869–78. doi: 10.1093/eurheartj/ehab493 (PMC8486488; doi:10.1093/eurheartj/ehab493)
Supplement: ehab493_Supplementary_Data [file ehab493_supplementary_data.zip › ehab493-suppl_data/3-tris-SupplTable1.docx]

**Supplementary Table 1 : ABCDE SE positivity by centers**

| **CENTER** | **Step A, n(%)** | **Step B, n(%)** | **Step C, n(%)** | **Step D, n(%)** | **Step E, n(%)** |
| --- | --- | --- | --- | --- | --- |
| **1 (n=5)** | 0 | 0 | 3 (60%) | 5 (100%) | 1 (20%) |
| **2 (n=18)** | 10 (55.6%) | 10 (55.6%) | 16 (88.9%) | 13 (72.2%) | 10 (55.6%) |
| **3 (n=1292)** | 59 (4.6%) | 247 (19.1%) | 202 (15.6%) | 167 (12.9%) | 387 (30%) |
| **4 (n=7)** | 1 (14.3%) | 2 (28.6%) | 7 (100%) | 5 (71.4%) | 6 (85.7%) |
| **5 (n=330)** | 28 (8.5%) | 53 (16.1%) | 215 (65.2%) | 151 (45.8%) | 111 (33.6%) |
| **6 (n=74)** | 10 (13.5%) | 10 (13.5%) | 62 (83.8%) | 13 (17.6%) | 29 (39.2%) |
| **7 (n=840)** | 384 (45.7%) | 397 (47.3%) | 475 (56.5%) | 388 (46.2%) | 487 (58.0%) |
| **8 (n=39)** | 11 (28.2%) | 7 (17.9%) | 21 (53.8%) | 18 (46.2%) | 3 (7.7%) |
| **9 (n=5)** | 0 | 3 (60%) | 4 (80%) | 5 (100%) | 2 (40%) |
| **10 (n=774)** | 27 (3.5%) | 320 (41.3%) | 135 (17.4%) | 175 (22.6%) | 225 (29.1%) |
| **11 (n=7)** | 1 (14.3%) | 0 | 5 (71.4%) | 4 (57.1%) | 5 (71.4%) |
| **12 (n=20)** | 7 (35%) | 6 (30%) | 4 (20%) | 9 (45%) | 7 (35%) |
| **13 (n=163)** | 32 (19.6%) | 17 (10.4%) | 146 (89.6%) | 41 (25.2%) | 47 (28.8%) |
