# Supplementary material for: Prognostic value of stress echocardiography assessed by the ABCDE protocol
Source: Eur Heart J. 2021 Aug 27;42(37):3869–78. doi: 10.1093/eurheartj/ehab493 (PMC8486488; doi:10.1093/eurheartj/ehab493)
Supplement: ehab493_Supplementary_Data [file ehab493_supplementary_data.zip › ehab493-suppl_data/Supplfile Table 2.SlStress modality by centers.docx]

**Supplementary Table 2: Stress modality by centers**

| **CENTER** | **1** | **2** | **3** | **4** | **5** | **6** | **7** | **8** | **9** | **10** | **11** | **12** | **13** |
| --- | --- | --- | --- | --- | --- | --- | --- | --- | --- | --- | --- | --- | --- |
| **Stress** |  |  |  |  |  |  |  |  |  |  |  |  |  |
| Supine bike | 5 | 0 | 0 | 6 | 31 | 0 | 840 | 0 | 1 | 4 | 0 | 0 | 6 |
| Treadmill | 0 | 0 | 1 | 0 | 0 | 64 | 0 | 0 | 0 | 0 | 0 | 0 | 0 |
| Dipy | 0 | 18 | 1210 | 0 | 77 | 0 | 0 | 0 | 4 | 770 | 7 | 20 | 6 |
| Adenosine | 0 | 0 | 0 | 0 | 0 | 0 | 0 | 39 | 0 | 0 | 0 | 0 | 0 |
| Dobu | 0 | 0 | 81 | 1 | 222 | 10 | 0 | 0 | 0 | 0 | 0 | 0 | 157 |

Dipy,dipyridamole; dobu, dobutamine
